# INSL3 stimulates spermatogonial differentiation in testis of adult zebrafish (*Danio rerio*)

**DOI:** 10.1007/s00441-015-2213-9

**Published:** 2015-06-16

**Authors:** L. H. C. Assis, D. Crespo, R. D. V. S. Morais, L. R. França, J. Bogerd, R. W. Schulz

**Affiliations:** Laboratory of Cellular Biology, Department of Morphology, Institute of Biological Sciences, Federal University of Minas Gerais, Belo Horizonte, MG Brazil; Reproductive Biology Group, Division of Developmental Biology, Department of Biology, Faculty of Science, Utrecht University, Kruyt Building, Room W-606 & 607, Padualaan 8, NL-3584 CH Utrecht, The Netherlands

**Keywords:** INSL3, Spermatogonial differentiation, Androgen release, Gene expression, Adult testis, Zebrafish

## Abstract

INSL3 (insulin-like peptide 3) is a relaxin peptide family member expressed by Leydig cells in the vertebrate testis. In mammals, INSL3 mediates testicular descent during embryogenesis but information on its function in adults is limited. In fish, the testes remain in the body cavity, although the *insl3* gene is still expressed, suggesting yet undiscovered, evolutionary older functions. Anti-Müllerian hormone (Amh), in addition to inhibiting spermatogonial differentiation and androgen release, inhibits the Fsh (follicle-stimulating hormone)-induced increase in *insl3* transcript levels in zebrafish testis. Therefore, the two growth factors might have antagonistic effects. We examine human INSL3 (hINSL3) effects on zebrafish germ cell proliferation/differentiation and androgen release by using a testis tissue culture system. hINSL3 increases the proliferation of type A undifferentiated (A_und_) but not of type A differentiating (A_diff_) spermatogonia, while reducing the proliferation of Sertoli cells associated with proliferating A_und_. Since the area occupied by A_und_ decreases and that of A_diff_ increases, we conclude that hINSL3 recruits A_und_ into differentiation; this is supported by the hINSL3-induced down-regulation of *nanos2* transcript levels, a marker of single A_und_ spermatogonia in zebrafish and other vertebrates. Pulse-chase experiments with a mitosis marker also indicate that hINSL3 promotes spermatogonial differentiation. However, hINSL3 does not modulate basal or Fsh-stimulated androgen release or growth factor transcript levels, including those of *amh*. Thus, hINSL3 seems to recruit A_und_ spermatogonia into differentiation, potentially mediating an Fsh effect on spermatogenesis.

## Introduction

Insulin-like peptide 3 (INSL3) is a relaxin peptide family member expressed in the male reproductive system by Leydig cells during fetal development and adult life. During embryonic development in mammals, INSL3 has a role that is crucial for the success of spermatogenesis in adult life (Kumagai et al. 2002). Knockout mice for INSL3 or its receptor RXFP2 show a cryptorchid phenotype, in which the testes remain inside the body cavity and fail to descend into the scrotum, since the gubernacula do not develop properly (Zimmermann et al. 1999; Nef and Parada 1999; Kumagai et al. 2002). However, although INSL3/RXFP2 are also expressed in the adult testis, information on potential functions in mature testis is incomplete. During the last decade, some studies have suggested a role for INSL3 in spermatogenesis (Kawamura et al. 2004; Pathirana et al. 2012). The effect of INSL3 on germ cell survival has been recorded in rat (Kawamura et al. 2004), whereas a more recent study reported no effects on germ cell apoptosis following RXFP2 ablation in mice (Huang et al. 2012). Nevertheless, testosterone release by cultured mouse Leydig cells increases in response to INSL3 (Pathirana et al. 2012), suggesting an autocrine role of this peptide as previously proposed after the localization of RXFP2 to Leydig cells (Anand-Ivell et al. 2006).

Although more information concerning relaxins has been obtained in the past few years, knowledge of their biological activity mostly comes from studies in mammals and little is known about their role in submammalian vertebrates such as teleost fish, a taxonomic group including nearly 50 % of all vertebrate species (Yegorov et al. 2014). Studies regarding Insl3, other relaxins and their receptors in teleost fish are limited to gene expression data (Good-Ávila et al. 2009; Yegorov et al. 2009; Good et al. 2012). Evidently, no testicular descent occurs in fish and studies directed to finding other, evolutionary older biological activities might also provide new leads for the respective activities of INSL3 in higher vertebrates.

In zebrafish (*Danio rerio*), for example, *insl3* gene expression has been localized to Leydig cells (Good-Ávila et al. 2009). Recent studies with recombinant zebrafish follicle-stimulating hormone (Fsh) have shown that the stimulation of zebrafish testis explants with Fsh increases testicular *insl3* mRNA levels, an effect not mediated by the steroidogenic activity of Fsh (García-López et al. 2010) but, instead, by a direct effect on Leydig cells that express both gonadotropins receptors in fish (García-López et al. 2010; Ohta et al. 2007; Chauvigné et al. 2012). On the other hand, recombinant anti-Müllerian hormone (Amh) inhibits the stimulatory effect of Fsh on *insl3* mRNA levels in zebrafish (Skaar et al. 2011). This opens up the possibility that, at sites with high levels of Amh, Fsh is less efficient at increasing levels of *insl3* mRNA in Leydig cells. Since, in addition to suppressing *insl3* mRNA expression, Amh inhibits the differentiation of type A undifferentiated (A_und_) spermatogonia and Fsh-stimulated steroidogenesis, we hypothesize that Insl3 stimulates germ cell differentiation and steroidogenesis. This hypothesis is tested as part of our broader goal to understand the effect of Insl3 on testis function.

## Materials and methods

### Animals

Adult male zebrafish were bred and raised in the aquarium facility of the Department Biology, Utrecht University. The experiments followed the Dutch National regulations for animal use in experimentation. For morphometric/androgen release and mRNA analyses, 8 and 12 animals were used per experiment, respectively.

### Human INSL3

Human INSL3 (hINSL3) was synthesized by using the continuous flow Fmoc (N-(9-fluorenyl)methoxycarbonyl)-solid phase methodology together with regioselective disulfide bond formation as previously described (Bathgate et al. 2006) and was obtained as a kind gift from Prof. John D. Wade, University of Melbourne, Victoria, Australia. The peptide was dissolved at a concentration of 100 μg/ml in sterile phosphate-buffered saline (PBS) and aliquots were flash-frozen in liquid N_2_ and stored at −80 °C. We reasoned that hINSL3 would be biologically active in zebrafish testis because of the following considerations. Two *rxfp2* genes (*rxfp2a* and *rxp2b*), paralogous to the human *RXFP2* gene, are abundantly expressed in zebrafish testis (Good et al. 2012; Yegorov et al. 2014). The specificity of the interaction of hINSL3 with RXFP2 is mainly determined by RXFP2 residues Phe^131^ and Gln^133^ interacting with hINSL3 B-chain residue Trp^27^, RXFP2 residue Trp^177^ with hINSL3 B-chain residue His^12^, RXFP2 residue Ile^179^ with hINSL3 B-chain residue Val^19^, RXFP2 residues Asp^181^ and Glu^229^ with hINSL3 B-chain residue Arg^20^ and Asp^227^ with hINSL3 residue Arg^16^ (Büllesbach and Schwabe 1999, 2004, 2005, 2006; Rosengren et al. 2006; Scott et al. 2007). Alignment of the zebrafish Rxfp2a and Rxfp2b receptor sequences with the human RXFP2 receptor sequence revealed that the zebrafish receptor contains identical residues at the ligand-receptor interaction sites, except for RXFP2 residues Ile^179^ and Glu^229^, which are replaced by Val and Ala in the zebrafish Rxfp2a receptor. Human INSL3 should therefore be able to interact with both zebrafish Rxfp2 receptors, as Val^179^ and Ala^229^ would not hinder hINSL3 interacting with the Rxfp2a receptor.

### Tissue culture

A primary testis tissue culture system was used to study the effects of hINSL3 on germ and somatic cell proliferation, androgen release and testicular mRNA levels, according to protocols previously established (Leal et al. 2009b). The concentration of 100 ng hINSL3/ml was chosen based on a pilot experiment and data published in mammals (Pathirana et al. 2012). To study proliferation and transcript levels, the two testes from each fish were dissected and incubated for 7 days, one under stimulatory conditions (receiving medium containing hINSL3) and the other under basal conditions (receiving only tissue culture medium). The testes were placed on a 0.25-cm^2^ piece of nitrocellulose membrane (25 μm thickness, 0.22 μm pore size; Millipore, Billerica, Mass., USA), on top of a 700-μl agarose cylinder (1.5 % [w/v] in Ringer’s solution, pH 7.4) that was placed in a 24-well flat-bottom plate (Corning, New York, USA). Medium (1 ml) was added such that the agar cylinder was just not submerged; the medium was refreshed after 4 days. To study androgen release, testes were submerged in 200 μl tissue culture medium in a 96-well plate for ~18 h. All components for the tissue culture studies were freshly prepared according to published protocols (Leal et al. 2009b).

### Germ and Sertoli cell proliferation analysis

To evaluate the capacity of hINSL3 to modulate the proliferation activity of various spermatogonial generations and of Leydig and Sertoli cells, 50 μg/ml proliferation marker 5-bromo-2′-deoxyuridine (BrdU; Sigma-Aldrich) was added to the tissue culture medium of the testes during the last 6 h of the 7-day incubation period. During this final period of 6 h, the tissue was submerged in the medium.

In a pulse-chase set-up, zebrafish were exposed in vivo to BrdU dissolved in water (4 mg/ml) for ~12 h per day on 3 consecutive days to allow BrdU incorporation into the DNA of all dividing cells, including slowly dividing, single type A_und_ spermatogonia, followed by a 4-day chase period, during which the BrdU labeling was cleared from rapidly dividing cell types, including many of the rapidly proliferating spermatogonia (Nóbrega et al. 2010). Subsequently, the testes were dissected and incubated for 4 days ex vivo in the absence or presence of hINSL3 (100 ng/ml), as described above. Importantly, BrdU was not present in the medium during this tissue culture period of 4 days, so that the localization of the proliferation marker at the end of the tissue culture period allowed hINSL3 effects on the dynamics of BrdU to be examined in the germ cells that had taken up the marker previously in vivo.

**Fig. 1:**
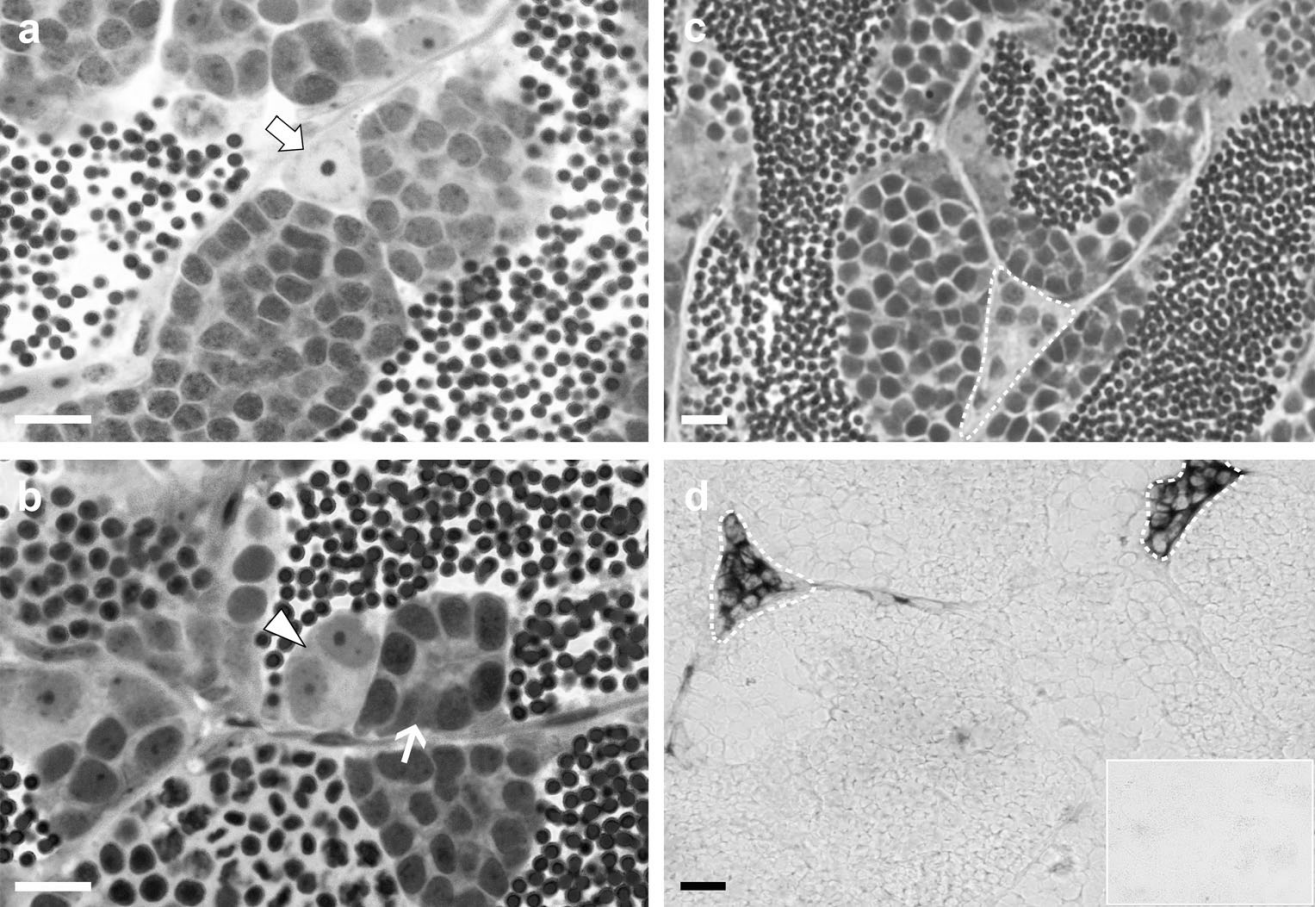
Morphological characteristics of zebrafish type A undifferentiated (A_und_) and type A differentiating (A_diff_) and type B spermatogonia and Leydig cells and in situ hybridization for *insl3* mRNA in adult zebrafish testis. **a** Type A_und_ spermatogonia (*arrow*) are single germ cells showing a large and clear nucleus, with poorly condensed chromatin and one or two compact nucleoli. **b** A cyst containing two type A_diff_ spermatogonia (*arrowhead*) that show smaller nuclei stained more intensely by toluidine-blue. Type B spermatogonia (*thin arrow*) have still smaller, slightly elongated/ovoid nuclei that moreover show a high amount of heterochromatin. **c** Interstitial space (*dashed line*) with a group of Leydig cells. **d** Adult zebrafish testis section showing the detection of *insl3* mRNA by in situ hybridization in Leydig cells cluster (*dashed lines*). *Inset* in **d** non-specific staining obtained with the sense probe. **a–c** Sections were prepared for morphological analysis according to Leal et al. (2009b). Magnification ×1000 (**a**, **b**), ×600 (**c**, **d**). *Bars* 10 μm

After the tissue culture period, the testes were fixed at 4 °C overnight in freshly prepared methacarn (60 % [v/v] absolute ethanol, 30 % chloroform and 10 % acetic acid), dehydrated, embedded in Technovit 7100 (Heraeus Kulzer), sectioned at a thickness of 4 μm and used to localize BrdU, as described previously (Leal et al. 2009a). The germ cells/cysts were identified according to previously published morphological criteria (Leal et al. 2009a). In brief (see Fig. 1a), the type A_und_ spermatogonium is a single germ cell and the largest spermatogonial cell type in zebrafish, with a large nucleus (~9 μm diameter), poorly condensed chromatin and one or two compact nucleoli; it is enveloped by a Sertoli cell, thus forming the initial stage of a spermatogenic cyst. The type A_diff_ spermatogonia, although morphologically not very different from type A_und_, show a smaller (~6 μm diameter) and denser nucleus and occur in groups of 2, 4, or 8 cells (1st, 2nd and 3rd/final generation of type A_diff_ spermatogonia) in the same stage of development inside a cyst (Fig. 1b), because of the synchronized development among the members of the same germ cell clone based on cytoplasmic bridges remaining from an incomplete cytokinesis during differentiating mitoses. The five generations of type B spermatogonia (16 to 256 cells) show a further reduced nuclear size (~5 μm diameter); the nucleus is slightly elongated/ovoid and clearly contains more heterochromatin than in type A spermatogonia (Fig. 1b).

To quantify proliferation, the mitotic index was determined by examining 100 randomly chosen germ cells/cysts or somatic cells and by discriminating between BrdU-labeled and unlabeled cells. To evaluate the proportion of area occupied by type A_und_, A_diff_ and B spermatogonia, 30 randomly chosen fields were photographed at ×400 magnification by using a conventional microscope equipped with a digital camera. The images were analyzed quantitatively by using ImageJ software (Image Processing and Analysis in Java). With a specific plug-in, a 540-point grid was made to quantify the proportion of the area for the various germ cell types, based on the number of points counted over those germ cell types.

### Relative quantification of testicular mRNA levels

The effects of hINSL3 on testicular mRNA levels were investigated after 7 days of testis tissue culture. Total RNA was isolated from the tissue by using an RNAqueous Micro kit (Ambion), according to the manufacturer’s protocol, in order to quantify the mRNA levels of selected testicular genes. The selection included *insl3* (García-López et al. 2010), Sertoli cell genes known to modulate spermatogonial proliferation and differentiation behavior, such as *amh* (Miura et al. 2002; Skaar et al. 2011), *gsdf* (Sawatari et al. 2007) and *igf3* (Morais et al. 2013) and four germ cell markers, namely *nanos2* (expressed in single type A_und_ spermatogonia; Beer and Draper 2013), *piwil1* (expressed in all generations of type A spermatogonia; Houwing et al. 2007), *sycp3* (expressed in spermatocytes; Chen et al. 2013) and *odf3b* (expressed in spermatids; Yano et al. 2008). Two micrograms of isolated RNA was taken from each sample to synthesize cDNA as described previously (de Waal et al. 2008). The relative mRNA levels were determined by using real time, quantitative polymerase chain reaction (qPCR) assays, according to published protocols for all genes analyzed (see Table 1), except for *nanos2*. For detecting *nanos2* mRNA (Beer and Draper 2013), primers were designed (Table 1) and validated for specificity and amplification efficiency on serial dilutions of testis cDNA (Bogerd et al. 2001). All qPCRs and calculations were performed as described previously (Bogerd et al. 2001; de Waal et al. 2008; García-López et al. 2010) in 20-μl reaction volumes and quantification cycle (Cq) values were obtained by a Step One Plus Real-Time PCR System (Applied Biosystems) by using default settings. Elongation factor 1α (*ef1α*) mRNA was used as the endogenous control, since its expression remained stable under both basal and treated conditions.

**Table 1:** Primers used for mRNA levels measurement of *ef1α* (elongation factor 1α), *insl3* (insulin-like peptide 3), *amh* (anti-Müllerian hormone), *gsdf, igf3* (two Sertoli cell genes known to modulate spermatogonial proliferation and differentiation), *nanos2, piwil1, sycp3, odf3b* (four germ cell markers); Fw, forward; Rv, reverse

Target genes	Primers	Sequence (5′-3′)	Reference
*ef1α*	AG (Fw)	GCCGTCCCACCGACAAG	Morais et al. 2013
	AH (Rv)	CCACACGACCCACAGGTACAG	
*insl3*	2466 (Fw)	TCGCATCGTGTGGGAGTTT	Good et al. 2012
	2467 (Rv)	TGCACAACGAGGTCTCTATCCA	
*amh*	AD (Fw)	CTCTGACCTTGATGAGCCTCATTT	García-López et al. 2010
	AE (Rv)	GGATGTCCCTTAAGAACTTTTGCA	
*igf3*	2680 (Fw)	TGTGCGGAGACAGAGGCTTT	Morais et al. 2013
	2681 (Rv)	CGCCGCACTTTCTTGGATT	
*gsdf*	2366 (Fw)	CATCTGCGGGAGTCATTGAAA	García-López et al. 2010
	2367 (Rv)	CAGAGTCCTCCGGCAAGCT	
*piwi1l*	2542 (Fw)	GATACCGCTGCTGGAAAAAGG	García-López et al. 2010
	2543 (Rv)	TGGTTCTCCAAGTGTGTCTTGC	
*sypc3*	2730 (Fw)	AGAAGCTGACCCAAGATCATTCC	García-López et al. 2010
	2731 (Rv)	AGCTTCAGTTGCTGGCGAAA	
*odf3b*	2791 (Fw)	GATGCCTGGAGACATGACCAA	Leal et al. 2009b
	2792 (Rv)	CAAAGGAGAAGCTGGGAGCTTT	
*nanos2*	4817 (Fw)	AAACGGAGAGACTGCGCAGAT	This paper
	4818 (Rv)	CGTCCGTCCCTTGCCTTT	

### Cellular localization of *insl3* mRNA

To localize the cellular expression of *insl3* mRNA, in situ hybridization was performed by using digoxigenin (DIG)-labeled cRNA probes previously designed and validated (Good-Ávila et al. 2009). Adult zebrafish testes were fixed in 4 % paraformaldehyde (PFA) in PBS (pH 7.4) at 4 °C overnight and subsequently transferred to 20 % sucrose in PBS until the tissue remained submerged. In situ hybridization was carried out on 10-μm-thick cryosections (Leica cryostat), which were post-fixed with 4 % PFA and then treated with proteinase K (5 μg/mL; Sigma-Aldrich) at room temperature for 5 min. Hybridization, with 500 ng riboprobe per milliliter, was carried out in hybridization buffer (5 × standard sodium citrate, 50 % deionized formamide, 10 % dextran sulfate, 5 × Denhardt’s, 250 μg/ml yeast tRNA, 500 μg calf thymus DNA) at 65 °C overnight. The slides were then washed in 5 × and 0.2 × standard sodium citrate at 55 °C, each for 30 min and blocking was performed with 1 % heat-inactivated goat serum (Vector) in blocking buffer (0.1 M TRIS–HCl, 0.15 M NaCl, pH 7.5) at room temperature for 1 h. Subsequently, the slides were incubated with alkaline-phosphatase-conjugated anti-DIG antibody (Roche) diluted 1:1000 in blocking solution at 4 °C overnight. Color development was conducted by incubating the sections in Nitroblue tetrazolium/5-bromo-4-chloro-3-indolylphosphate (Roche) in the dark. After being air-dried, the slides were mounted using Aquamount (Thermo Scientific) and micrographs were taken with an Olympus AX70 microscope.

### Androgen release analysis

To determine whether basal or Fsh-stimulated testicular androgen release was modulated by hINSL3, the levels of 11β-hydroxyandrostenedione (OHA), a known precursor of 11-ketotestosterone (11-KT), were measured in testis tissue culture medium using a steroid release bioassay previously adapted for zebrafish testis (García-López et al. 2010). The results were calculated as nanograms of OHA released per milligram of testis tissue when incubated in the absence or presence of 25 ng/ml Fsh and/or 100 ng/ml hINSL3, respectively.

### Statistics

The statistical analyses were performed by using the GraphPad Prism 5 software package (GraphPad Software, San Diego, Calif., USA). Differences between control and experimental groups were tested for statistical significance by using the Student’s *t*-test for paired observation and analysis of variance (post-test Newman-Keuls) for multiple group comparisons. A significance level of *P* < 0.05 was applied in all analyses. Data are presented as means ± standard error of mean (SEM).

## Results

### Spermatogonial and somatic cell proliferation

**Fig. 2:**
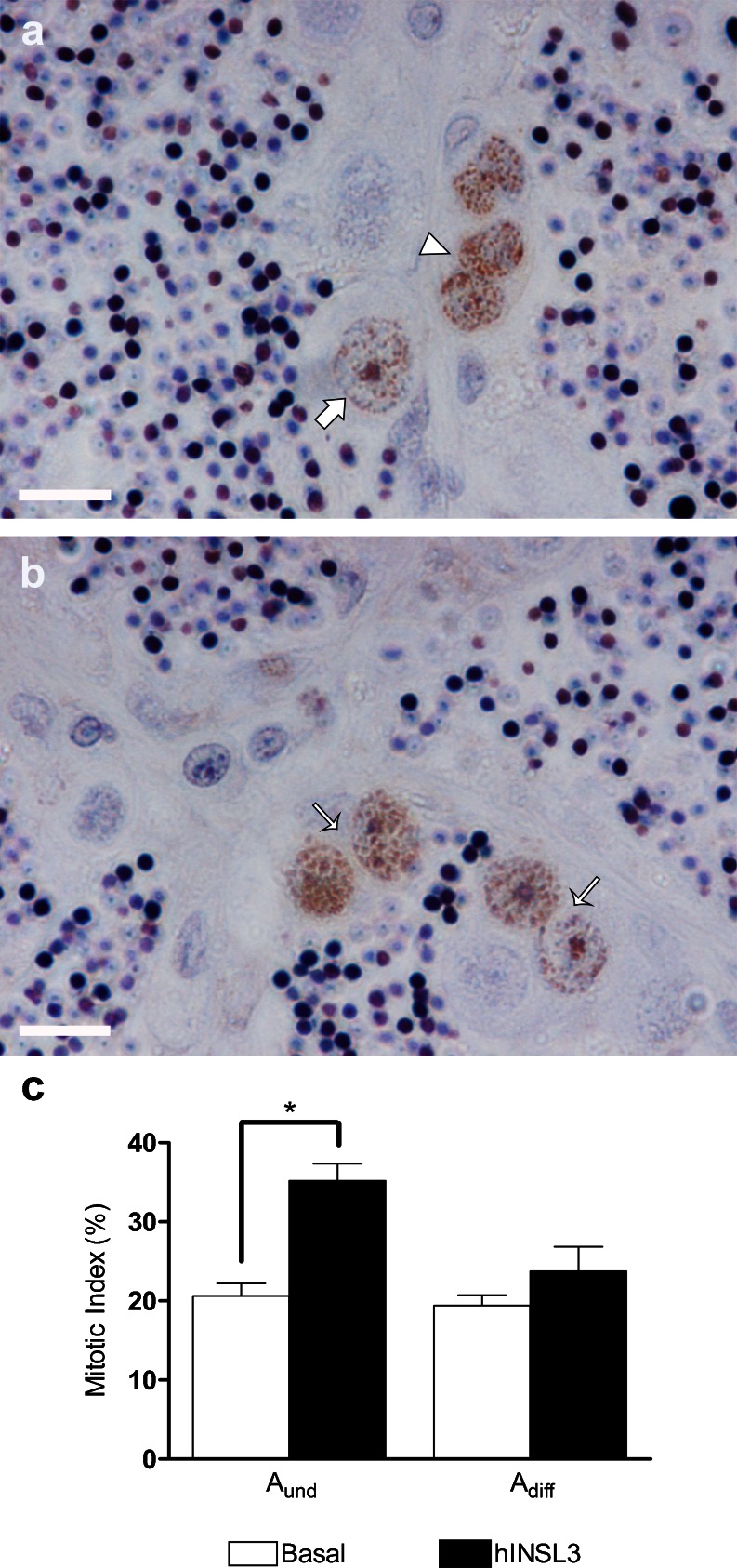
**a**, **b** Testis tissue sections showing BrdU-positive germ cells: type A_und_ (*arrow*), type A_diff_ (*thin arrows*) and type B (*arrowhead*) spermatogonia. Magnification ×1000. *Bars* 10 μm. **c** Mitotic indices of type A_und_ and A_diff_ spermatogonia after incubation for 7 days in the absence (*Basal*) and presence (*hINSL3*) of 100 ng hINSL3/ml. *Significant difference (*P* < 0.05) between treated and control. Results are presented as means ± SEM (*n* = 8)

**Fig. 3:**
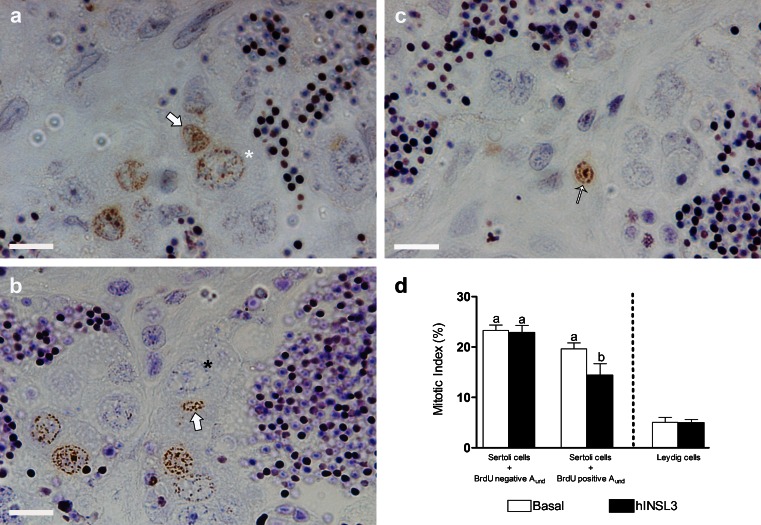
**a**, **b** Testis tissue sections showing BrdU-positive Sertoli cells nuclei (*arrows*) in association with BrdU-positive (*white star*) and BrdU-negative (*black star*) type A_und_ spermatogonia. **c** BrdU-positive Leydig cell nucleus (*thin arrow*). **d** Mitotic indices of Sertoli cells in association with BrdU-negative or BrdU-positive type A_und_ spermatogonia and of Leydig cells after incubation for 7 days in the absence (*Basal*) or presence (*hINSL3*) of 100 ng hINSL3/ml. Different *letters* indicate significant differences (*P* < 0.05) between the absence and presence of hINSL3. Magnification ×1000 (**a–c**). *Bars* 10 μm. Results are presented as means ± SEM (*n* = 8)

Based on counting BrdU-positive cells, we calculated the mitotic index of type A_und_ and A_diff_ spermatogonia (Fig. 2a, b), Leydig cells and Sertoli cells associated with BrdU-positive or BrdU-negative type A_und_ spermatogonia (Fig. 3a-c). In testis tissue culture, hINSL3 significantly stimulated the proliferation of type A_und_ spermatogonia, whereas no significant changes were observed for type A_diff_ spermatogonia (Fig. 2c). Moreover, no effects were observed for Leydig cells or for Sertoli cells associated with BrdU-negative type A_und_ spermatogonia, whereas a significant decrease in proliferation was found for Sertoli cells associated with BrdU-positive type A_und_ spermatogonia (Fig. 3d).

**Fig. 4:**
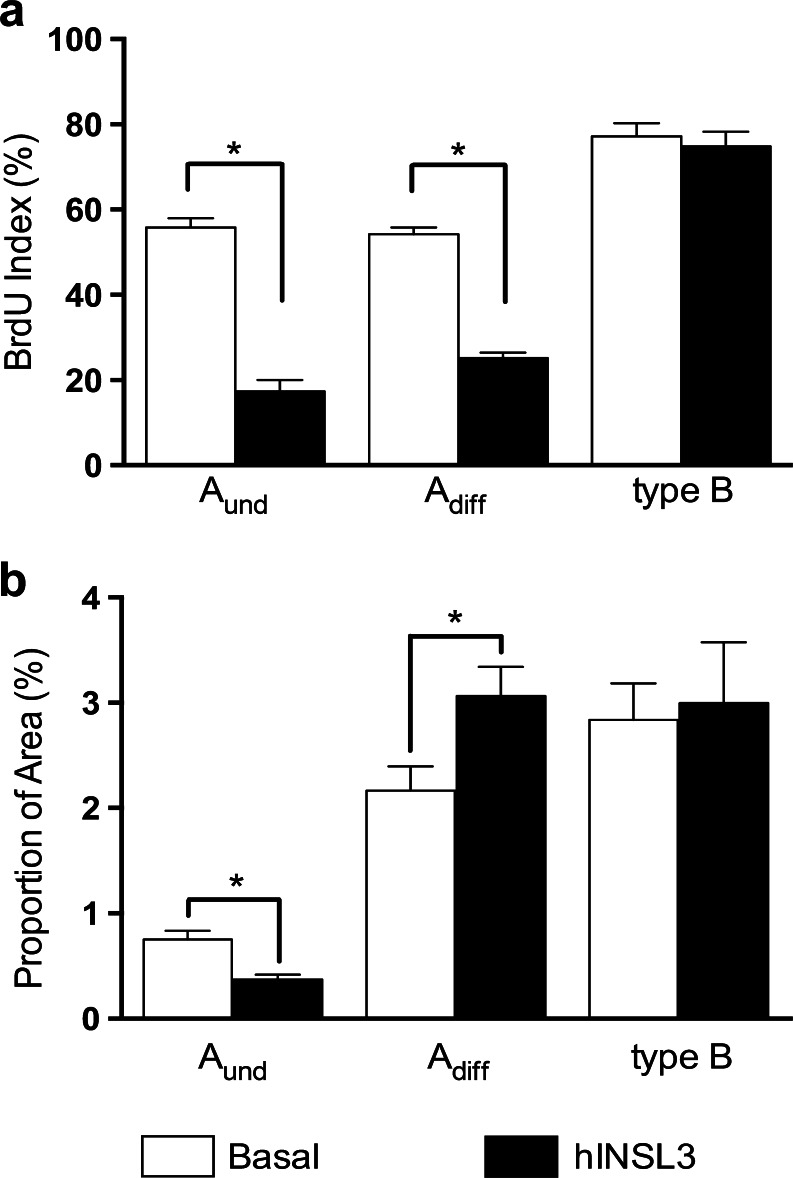
BrdU indices of type A_und_, type A_diff_ and type B spermatogonia after BrdU exposure in vivo and subsequent tissue culture in the absence (*Basal*) and presence (*hINSL3*) of 100 ng hINSL3/ml (**a**) and volumetric proportion of cysts of type A_und_, type A_diff_, and type B spermatogonia after tissue culture in the absence (*Basal*) and presence (*hINSL3*) of hINSL3 (**b**). *Significant differences (*P* < 0.05) between the absence and presence of hINSL3. Results are presented as means ± SEM (*n* = 8)

In order to determine whether the increased proliferation activity of type A_und_ spermatogonia was associated with self-renewal (leading to two single A_und_) or differentiation (leading to a pair of A_diff_), we labeled these cells with BrdU in vivo via a 3-day pulse and 4-day chase protocol, before the exposure of testis tissue to hINSL3 ex vivo. This allowed the study of the dynamics of spermatogonial proliferation by examining the way that exposure to hINSL3 ex vivo influenced the BrdU indices of type A_und_, type A_diff_ and type B spermatogonia that previously had taken up the BrdU label in vivo. Quantification of the BrdU indices in this type of experiment for the various spermatogonial cell types showed that hINSL3 induced a decrease of approximately three- to two-fold, respectively, for type A_und_ and A_diff_ spermatogonia, whereas no change was recorded for type B spermatogonia (Fig. 4a). At the same time, the proportion of the area occupied by type A_und_ spermatogonia was significantly reduced, whereas an increase was observed in the proportion of type A_diff_ spermatogonia (Fig. 4b).

### Androgen release

**Fig. 5:**
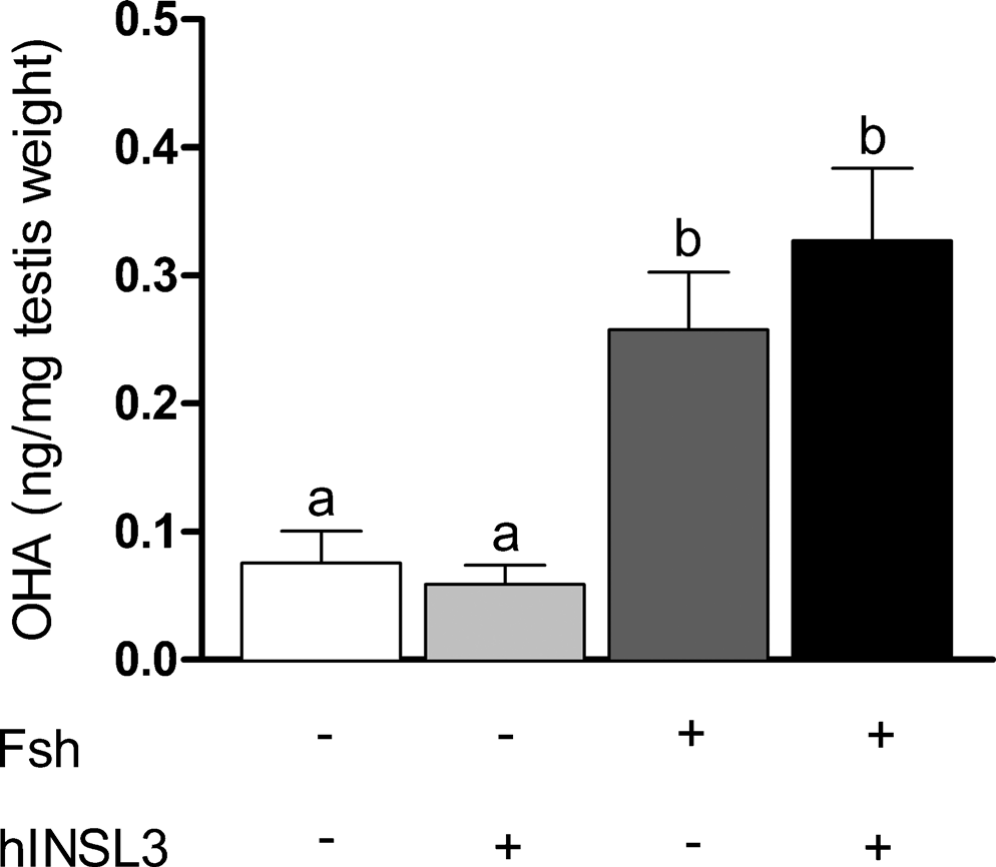
Effects of hINSL3 (100 ng/ml) on basal or follicle-stimulating hormone (*Fsh*; 25 ng/ml)-stimulated androgen release ex vivo. Results (means ± SEM; *n* = 8) are presented as nanograms of 11β-hydroxyandrostenedione (*OHA*) released per milligram (mg) of testis weight

To evaluate whether the effects of hINSL3 on germ cell proliferation and differentiation were associated with a modulation of basal or gonadotropin-stimulated androgen release, we used (recombinant zebrafish) Fsh, which is a potent steroidogenic hormone in fish (reviewed by Schulz et al. 2010). Our results showed that hINSL3 neither modulated basal nor Fsh-stimulated androgen release (Fig. 5).

### Relative testicular transcript levels and cellular localization of *insl3* mRNA

**Fig. 6:**
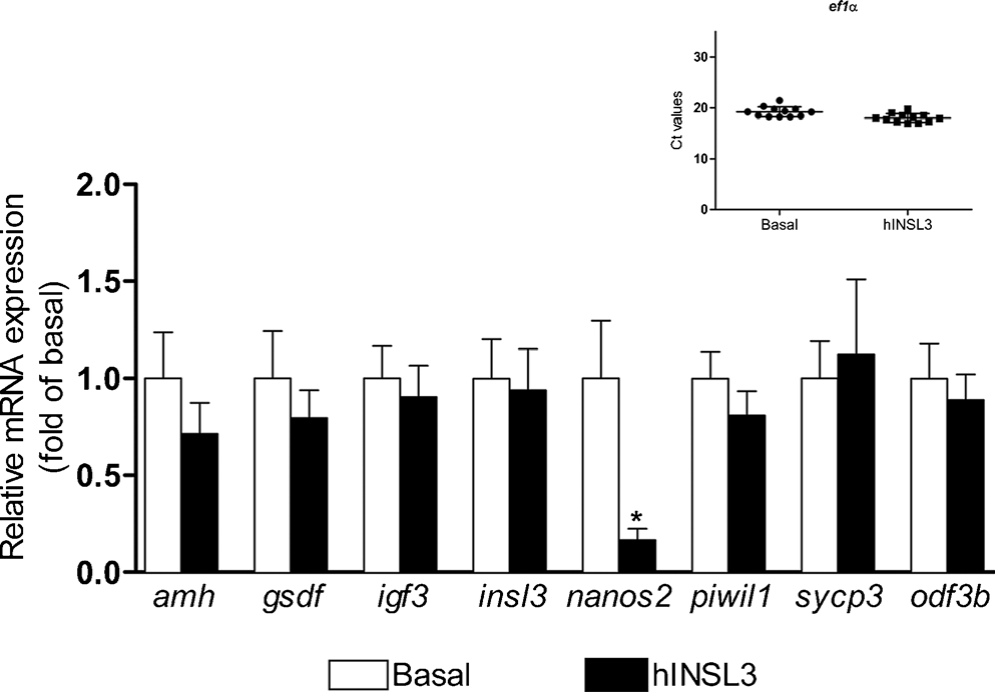
Relative mRNA levels of insl3 and selected Sertoli (*amh*, *gsdf* and *igf3*) and germ cell (*nanos2*, *piwil1*, *sypc3* and *odf3b*) genes. *Inset* Transcript levels of *ef1α* in the absence (*Basal*) and presence (*hINSL3*) of hINSL3; Ct values for the reference gene *ef1α*. Results are presented as means ± SEM (*n* = 12)

The quantification of the relative transcript levels of selected genes was intended to start an elucidation of the molecular mechanism used by hINSL3 to modulate germ cell proliferation. We analyzed the transcript levels of *insl3*, three Sertoli cell genes (*amh*, *gsdf* and *igf3*) and a spermatogonial (*piwil1*), a spermatocyte (*sycp3*) and a spermatid (*odf3b*) gene. The qPCR results showed no significant difference between basal and hINSL3-stimulated conditions for these transcripts (Fig. 6). However, transcript levels of *nanos2*, a marker for single type A_und_ spermatogonia, was down-regulated six-fold in testis tissue exposed to hINSL3 (Fig. 6).

The cellular localization of *insl3* transcripts in zebrafish testis tissue was analyzed by in situ hybridization on cryosections. Transcripts were exclusively observed in the interstitial compartment (Fig. 1d), with a specific hybridization signal exclusively on Leydig cells that often formed clusters in the interstitium (Fig. 1c, d). No signal was observed with the sense *insl3* riboprobe (negative control; inset Fig. 1d).

## Discussion

Although information is available concerning the evolution and expression of relaxin peptide family members in teleost fish (Good-Ávila et al. 2009; Yegorov et al. 2009; Good et al. 2012), no previous reports exist, to our knowledge, on the biological activity of these peptides in submammalian vertebrates. We reasoned that hINSL3 would be able to interact with both zebrafish Rxfp2 receptors, since the residues identified as important for ligand-receptor interaction with the human receptor for hINSL3 (i.e., RXFP2) are identical in the zebrafish Rxfp2a and Rxfp2b receptors, except for RXFP2 residues Ile^179^ and Glu^229^, which are replaced by Val and Ala and most likely do not hinder the interaction of hINSL3 with the zebrafish Rxfp2a receptor. This is supported by our finding that hINSL3 stimulates the differentiation of type A_und_ spermatogonia (further discussed below). Hence, despite the biological differences in the action of INSL3 in teleosts vs. mammals and their evolutionary distance, paralogous teleost receptors, Rxfp2a and Rxfp2b, are still able to respond to mammalian hINSL3. This suggests that, although the neofunctionalization of certain aspects of INSL3 occurs in mammals (i.e., testicular descent), older conserved functions of INSL3 presumably take place across vertebrates. Whereas spermatogenesis is known not to be dependent on INSL3 function in adult mice (Huang et al. 2012), INSL3 might have as yet to be identified effects on germ cell development in higher vertebrates and the detection of RXFP2 in mammalian germ cells is compatible with the notion that INSL3 modulates germ cell development in a paracrine manner (Huang et al. 2012; Minagawa et al. 2014; Sagata et al. 2015), notwithstanding the known (neo-functionalized) role of INSL3 in mammalian testicular descent.

Using a testis tissue culture approach, we showed that hINSL3 significantly increases the mitotic index of type A_und_ spermatogonia. Moreover, we propose that these proliferating type A_und_ spermatogonia are preferentially recruited into differentiation by hINSL3. Type A_und_ spermatogonia are single germ cells enveloped by one or sometimes two Sertoli cells in zebrafish (Leal et al. 2009a); spermatogonial stem cells (SSC) are part of this germ cell population (Nóbrega et al. 2010). As undifferentiated cells, SSCs have the capacity to produce either more SSCs (self-renewal proliferation) or germ cells committed to the spermatogenic process (differentiating proliferation), the first step being to form a pair of considerably smaller type A_diff_ spermatogonia. The latter cells represent ~44 % of the cellular volume of A_und_, such that a pair of A_diff_ are slightly smaller than a single A_und_ (Leal et al. 2009a) and remain connected via a cytoplasmic bridge inside a single cyst, one of the hallmarks of differentiating germ cell divisions. On the other hand, SSC undergoing self-renewal produce two completely separated type A_und_ daughter cells and the newly generated germ cell needs to recruit its own Sertoli cell to create a new spermatogenic cyst. Consequently, to produce new spermatogenic cysts, Sertoli cell proliferation is also required (França et al. 2015). Here, we found that Sertoli cells contacting BrdU-positive type A_und_ spermatogonia show reduced proliferation activity when incubated with hINSL3 (Fig. 3d), which we interpret as circumstantial evidence that the A_und_ spermatogonia are undergoing germ cell differentiation not self-renewal. To obtain more direct evidence for this hypothesis, we used additional molecular and morphological approaches.

Quantifying selected germ cell marker genes show that three out of the four transcripts remain stable, whereas *nanos2* mRNA levels are down-regulated in testis tissue exposed to hINSL3. In male mice, SSC maintenance depends on the RNA binding protein NANOS2; its conditional loss in adults results in a loss of SSCs to differentiation, whereas overexpression increases testicular SSC numbers (Sada et al. 2009). Furthermore, miR-34c-mediated down-regulation of the NANOS2 protein enhances murine SSC differentiation (Yu et al. 2014). In teleost fish, Nanos2 appears to play a similar role, as the loss of *nanos2* function in tilapia results in germ-cell-deficient testes (Li et al. 2014) and as both protein and transcript are detected in single type A_und_ spermatogonia (Lacerda et al. 2013; Bellaiche et al. 2014). In the adult zebrafish testis, *nanos2* mRNA is expressed exclusively in single, *vasa*-positive germ cells that are considered to have germ line stem cell-like characteristics in both sexes (Beer and Draper 2013). Hence, a decrease in *nanos2* transcript levels in testis tissue incubated with hINSL3 is compatible with a hINSL3-induced differentiation of single type A_und_ spermatogonia, such as the SSCs in zebrafish.

A second approach that we employed is based on quantitative histology. In agreement with the decreased *nanos2* transcript levels, we found a decrease in the area occupied by type A_und_ spermatogonia and an increased area occupied by A_diff_ spermatogonia (Fig. 4b). Hence, this increase in type A_diff_ cells directly suggests that hINSL3 stimulation increases the proliferation of A_und_ spermatogonia and their differentiation into A_diff_ spermatogonia but does not change the mitotic activity of A_diff_ spermatogonia.

We observed that A_und_ and A_diff_ spermatogonia pre-labeled with BrdU in vivo lose the BrdU label faster during a subsequent tissue culture period of 4 days when incubated in the presence but not absence of hINSL3 (Fig. 4a). We consider this to be evidence that hINSL3 is involved in the differentiation of type A_und_ spermatogonia. To explain this, we need to refer to spermatogonial dynamics in zebrafish.

Tracing a BrdU pulse through time, Leal et al. (2009a, 2009b) demonstrated that meiosis and spermiogenesis in zebrafish take 6 days, both in vivo and in tissue culture. The only information available for the duration of the mitotic phase of zebrafish germ cell differentiation indicates that one cell cycle of type A_und_ spermatogonia takes place within 30 h (Nóbrega et al. 2010). Therefore, we estimate that, during the 96 h of tissue culture for the experiments shown in Fig. 4, three to four spermatogonial divisions might have taken place. Since BrdU is incorporated into the A_und_ spermatogonia at the beginning of the tissue culture, this means that, in the case of differentiating divisions, the BrdU would have shifted into the A_diff_ cell pool, leading to a decrease of the BrdU index for type A_und_ cells. Thus, in conjunction with the reduction of *nanos2* transcript levels, the surface area occupied by A_und_ spermatogonia (Fig. 4b) and the loss of BrdU from the A_und_ cell pool is the third line of evidence showing that hINSL3 induces the differentiation of single type A_und_ spermatogonia in zebrafish.

However, the BrdU index of the A_diff_ spermatogonia is also significantly reduced following exposure to hINSL3 (Fig. 4a). This is unexpected, since their proliferation activity is not changed by hINSL3 (Fig. 2c) but might be explained by the following considerations. The hINSL3-mediated recruitment of type A_und_ cells into differentiation probably occurs irrespective of their BrdU-labeling status. About 45 % of the A_und_ spermatogonia do not contain BrdU under control conditions (Fig. 4a: 55 % BrdU-labeled, i.e., 45 % unlabeled) and hINSL3 will have stimulated the production of BrdU-negative A_diff_ from the initially BrdU-negative A_und_ spermatogonia. Moreover, initially, BrdU-positive type A_und_ spermatogonia recruited by hINSL3 into differentiation potentially undergo three or four cell cycles (three cycles from A_und_ to the third and last generation of A_diff_, or four cycles to the first generation of type B spermatogonia), which is associated with an 8- to 16-fold dilution of BrdU that thereby might have become undetectable. We propose that the reduced BrdU index of type A_diff_ spermatogonia, despite their unchanged mitotic activity, reflects the combined effects of an increased production of BrdU-negative A_diff_ cells from initially BrdU-negative A_und_ cells, a loss of BrdU detectability in A_diff_ cells by dilution attributable to repeated cell cycling and finally a shift of the BrdU label into the B spermatogonia population for the cells that undergo four differentiating cell cycles. Since the proportion of area occupied by A_diff_ spermatogonia becomes more prominent, although no change is observed for type B spermatogonia (Fig. 4b), we assume that the increased production of type A_diff_ with undetectable levels of BrdU is the main factor in this regard.

Since type B spermatogonia do not show changes after hINSL3 treatment for the parameters investigated (Fig. 4a, b), other experiments need to be performed to determine whether type B spermatogonia do not respond to hINSL3 or whether potential changes among type B spermatogonia are compensated for by the dynamics of “neighboring” germ cell generations (i.e., type A_diff_ spermatogonia and primary spermatocytes). However, major changes in the meiotic or post-meiotic germ cell generations do not seem likely considering the relatively stable expression levels of the marker genes *sypc3* and *odf3b*, respectively (Fig. 6).

Previous work has shown that Sertoli cell-derived Amh inhibits spermatogonial differentiation and also reduces Leydig cell *insl3* mRNA levels (Skaar et al. 2011). Since our present data suggest that hINSL3 stimulates spermatogonial differentiation, we hypothesize that hINSL3 action might include a down-regulation of *amh* transcript levels. However, this is not the case. Moreover, transcript levels of *gsdf* and *igf3*, coding for growth factors stimulating spermatogonial proliferation in trout (Sawatari et al. 2007) or zebrafish (Morais et al. 2013), are also unaffected by hINSL3, suggesting that a change in the transcript levels of these growth factors is not directly involved in mediating hINSL3 action.

Recent work in mice has shown that INSL3 has an autocrine stimulatory effect on androgen release from Leydig cells (Pathirana et al. 2012) and androgens are known to stimulate spermatogenesis in tissue culture in various fish species (e.g., Miura et al. 1991; Leal et al. 2009b). However, our results show no effect of hINSL3 on basal or Fsh-stimulated androgen release, indicating that hINSL3 effects on zebrafish spermatogonia are not mediated by acutely modulating androgen production.

In summary, in this work, we investigated the potential role of hINSL3 on spermatogenesis in zebrafish, a species in which *insl3* gene expression is also found in Leydig cells but in which testicular descent does not occur. Morphometric studies and gene analysis of germ cell markers support the hypothesis that hINSL3 stimulates the differentiating proliferation of type A_und_ to type A_diff_ spermatogonia. To our knowledge, this is the first study of the biological activity of a relaxin family member in fish reproduction. Future studies should be directed (1) at investigating whether zebrafish Insl3 shows comparable biological activities and (2) at identifying the testicular cell types expressing receptors for Insl3, as these aspects will lead us towards our larger goal of understanding the effects of Insl3 on testis function in teleosts.
